# Assessing the Information Potential of MIR Spectral Signatures for Prediction of Multiple Soil Properties Based on Data from the AfSIS Phase I Project

**DOI:** 10.3390/ijerph192215210

**Published:** 2022-11-17

**Authors:** Stanisław Gruszczyński, Wojciech Gruszczyński

**Affiliations:** AGH University of Science and Technology, Faculty of Geo-Data Science, Geodesy and Environmental Engineering, Al. Mickiewicza 30, 30-059 Krakow, Poland

**Keywords:** mid-infrared spectrum, soil properties prediction, partial least squares regression, 1D convolutional neural network, generalized regression neural network

## Abstract

The aim of the study was to assess the predictive potential of mid-infrared (MIR) spectral response in the estimation of 60 soil properties. It is important to know the accuracy limitations in estimating various soil characteristics using various models in conditions of high spatial variability of the environment. To fully assess this potential, three types of algorithms were used in modeling, i.e., partial least squares (PLSR), one-dimensional convolutional neural network (1DCNN), and generalized regression neural network (GRNN). The research used data from 19 sub-Saharan African countries collected as part of the Africa Soil Information Service (AfSIS) Phase I project. The repositories provide 18,250 MIR reflectance recordings and nearly two thousand analytical data records from the determination of many soil properties by reference methods. The modeled subset of these properties included texture (three variables), bulk density, moisture content at soil water characteristic curves (SWCC, 4 variables), total and organic C and total N content (3 variables), total elemental content (32 variables), elemental content in bioavailable forms (12 variables), electrical conductivity, exchangeable acidity, exchangeable bases, pH, and phosphorus sorption index. It is not possible to indicate a universal optimal prediction model for all soil variables. The best prediction results are provided by all regression models for total and organic C, total Fe, total Al and bioavailable Al content, and pH. For bulk density, total N and total K content satisfactory results are provided by specific model type. Many other properties, i.e., texture, SWCC, total Ga, Rb, Na, Ca, Cu, Pb, Hg content, and bioavailable Ca and K content, can be predicted with accuracies sufficient for some less demanding tasks.

## 1. Introduction

The interpretation of short-range spectral recordings under field or laboratory conditions attracts a lot of attention [1,2,3,4]. The importance of such methods is related to the three-dimensional variability of soils and their profile variation, which are impossible to recognize by recording spectral reflectance of the ground surface. Most studies to date relating to spectral analysis of soils have used the visible near-infrared (Vis-NIR) range covering the 350–2500 nm range of electromagnetic radiation [3,5,6]. Currently, there is increasing interest in investigating the suitability of the mid-infrared (MIR) spectrum covering the 2500–25,000 nm range [7,8,9].

The analysis of the usefulness of MIR signatures in soil research is of increasing interest. Margenot et al. analyzed the technical aspects of using this spectral range in the estimation of soil properties, mainly on the basis of a literature review [10]. They indicate the need to standardize the method of considering the impact of soil moisture on disturbances in the estimation of soil organic matter (SOM). Nath et al. analyze the usefulness of MIR signatures in estimating biological properties [11], pointing to the high usefulness of this approach in determining the concentration of C and N. They believe that the estimation of the concentration of enzymes and other organic components requires more sophisticated estimation methods. In a study of alfisols in eastern India [12], using a relatively small (approximately 330 samples) sample set, the authors concluded that the PLSR and SVM model provide a better estimate of SOC, pH, and clay than the random forest model. They point to the need to strengthen these conclusions on the basis of more territorially extensive research. An extensive analysis of the estimates of over 100 soil properties, based on the MIR Kellogg Laboratory’s spectral library, is contained in the publication by Ng et al. [8]. The results obtained confirmed the usefulness of MBL models for the estimation of many properties of soils from the territory of the USA.

A very insightful analysis of prediction results using NIR and MIR was conducted by Bellon-Maurel and McBratney [5]. The review analyzed the effect of the method of measurement implementation, data preprocessing, and algorithms used on soil organic carbon (SOC) prediction. According to one of the review’s conclusions, NIR and MIR allow the prediction of SOC with similar accuracy, with a slight advantage to MIR as it provides better reproducibility, with a lesser error (10–40% when compared to NIR). In addition, it was found that, regardless of the range of the spectrum, homogeneity of data for calibration and validation is essential. It should be noted that comparison of modeling results based on spectral response is justified only for soil samples with similar properties, geology, and climatic conditions. The importance of the range of variability of the data, the number of cases for calibration, preprocessing, and the type of model should also be emphasized.

In order to use spectral analysis in the soil survey, it is necessary to know the potential possibilities and limitations of this approach in estimating the values of various soil characteristics. The problem is complex. The model error, apart from its architecture, depends on the distribution and differentiation of data, the range of their variability, the number of observations, the method of validation, etc. The work of Nduwamungu et al. [13] summarizes the results of modeling tests based on the NIR spectrum of soil properties such as texture, SOC, pH, and cation exchange capacity (CEC) presented in the work of many authors, whose statistics differed dramatically: the *R*^2^ spread was between 0.01 and 0.99. The availability of possibly extensive data with high variability increases the credibility of the information potential of spectral data under various soil conditions. The knowledge of the potential usefulness of a spectrum for determining various soil properties is of significant practical importance for the selection of variables used to estimate soil quality indices [14,15].

Soils are described by a number of properties subject to evaluation from the point of view of their cultivation, economic, and engineering suitability. As evaluation criteria, depending on the use under consideration, various properties of soils are treated as more or less important. Classification of soils, in the context of their use in agriculture or forestry, requires characterization of basic physical (texture, water holding capacity, compactness, permeability, and many other potential physical indicators) and chemical (concentration of macro- and micronutrients, pH, sorption capacity, buffering capacity, and many others) properties. The list of elements to be assessed is constantly growing; for example, by including rare earth elements in the assessment of soils [16,17]—important because of their role as micronutrients and potential pollutants. Given this fact, it is important to appreciate the possibility of assessing the information potential contained in the spectral response of soil samples as a source to support, at least roughly, the assessment of environmentally or economically significant properties. It is important to note that the quality of models linking the spectral response to individual soil properties depends largely on the type of prediction algorithm used as well as the statistical distribution of the modeled properties. For this reason, it is advantageous to confront modeling approaches and validation datasets in order to identify features, prediction methods, and conditions for their use.

The aim of this research is to determine prediction accuracy for many soil variables based on the analysis of the spectral response of soils in the proximal sensing mode in the MIR range. The literature generally presents attempts to model several variables—usually C, N, P, K, pH, and texture, contents of some heavy metals, such as Zn, Hg, Cr, Cu, and Pb [18,19]—and occasionally other properties, such as soilwater characteristics curve, CEC, EC, or exchangeable bases [8]. AfSIS data are characterized by a very extensive list of variables marked with reference methods along with the spectral response [20]. They allow estimation of the modeling errors of many soil variables from samples taken in highly varied soil and climatic conditions. Determined validation statistics allow for the assessment of the usefulness and limitations of MIR models and data for estimating the values of variables characterizing soils.

## 2. Materials and Methods

### 2.1. AfSiS Phase I Data

AfSIS Phase I data were collected as part of a project financed by the Melinda and Bill Gates Foundation, implemented with the participation of five research institutions: The Tropical Soil Biology and Fertility Institute of The International Center for Tropical Agriculture, The International Soil Reference and Information Center—World Soil Information, The Center for International Earth Science Information Network, The Earth Institute at Columbia University, and World Agroforestry (Center for Research in Agroforestry-ICRAF). These data include the results of analyses of samples from soil profiles collected at points determined according to the methodology of the Land Degradation Surveillance Framework [20] in 19 countries of sub-Saharan Africa: Angola, Botswana, Burkina Faso, Cameroon, Ethiopia, Ghana, Guinea, Kenya, Madagascar, Malawi, Mali, Mozambique, Niger, Nigeria, South Africa, Tanzania, Uganda, Zambia, and Zimbabwe. More details regarding the AfSIS database can be found in Vågen et al. [21].

**Figure 1:**
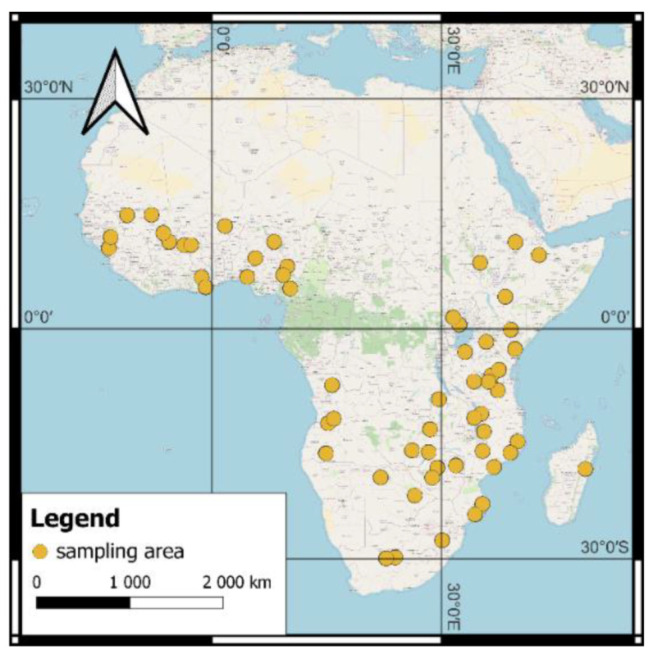
Locations of sampling areas in the AfSIS Phase I project. In each sampling area, a dozen sampling points were selected and the samples were taken from topsoil and subsoil layers.

The data provided by the repositories (Amazon AWS, GitHub, and the Agroforestry portal) came from topsoil (0–20 cm) and subsoil (20–50 cm) layers. Soil sampling was carried out in accordance with the Land Degradation Surveillance Framework (LDSF) procedure, taking into account the local and spatial characteristics of the environment. The sampling areas represent a random stratification of the African landscapes south of the Sahara desert (Figure 1). The samples were taken in the areas surrounding the cluster center, which allowed consideration of the spatial variability of soils [22]. A detailed method for determining the location of sampling areas, sampling, and analytical procedures is included in the report written by Leenaars et al. [23].

AfSIS data were the basis for studies on the analysis of the spatial variability of soil chemical composition [24] and the characteristics of soil profiles [23]. On this foundation, cartographic documentation of soils with a resolution of 30 m was developed and prepared [25]. The repositories provide 18,250 MIR reflectance recordings [26,27]. A single spectral signature covers 1749 recorded reflectance values in the range from 2500 to 16,666 nm (wavelength 4000 to 600 cm^−1^ with a step of 1.9 cm^−1^). The average density of the spectral registration points was about 8.1 nm, and it ranged from 1.2 nm to 53 nm. The GitHub repository also provides nearly two thousand analytical data records from determinations of soil samples by reference methods. Georeferenced data on the location of sampling points are also available. The analytical data of the original dataset are split at the highest level into two groups, i.e., DryChemistry and WetChemistry. The DryChemistry subset includes the results of X-ray determinations of the content of 32 elements. The WetChemistry subset [28] includes data, which include soil texture, water characteristics of samples, and many soil chemical properties associated with the use of the Mehlich 3 methodology [29], from three different laboratories.

There are slight variations in the amount of data from different laboratories, which resulted in the need for separate modeling for individual parts of the set. Modeling of chemical data for which the reference data were less than 500 records were omitted. In addition, properties for which there are strict relationships allowing for their direct calculation on the basis of the modeled values were omitted in the modeling. Moreover, modeling of soil organic N was not carried out, as the method of determination of reference values was different from the commonly accepted standard. Each of the group of features for which modeling was performed included at least 1860 soil samples data, of which a randomly selected 70% was used for training and the remaining 30% was used for validation. The training and validation sets were kept the same for each model throughout the study.

A total of 60 variables were modeled, with the data divided into groups:

“Texture” (3 variables, 1307 training, and 560 validation samples), separated into three particle-size fractions, i.e., sand, silt, or clay;

“Water” (5 variables, 1306 training, and 560 validation samples), i.e., saturation at 0 (Sat. at 0), pF2.0, pF2.5, pF4.2, and bulk density (B. Den.);

“CN” (3 variables, 1331 training, and 567 validation samples): total N, total C, and SOC;

“Elements” (32 variables, 1333 training, and 570 validation samples), i.e., concentrations of: Na, Al, Cl, K, Ca, Ti, V, Cr, Mn, Fe, Ni, Cu, Zn, Ga, Se, Rb, Sr, Y, Zr, Ba, La, Ce, Pr, Nd, Sm, Hf, Ta, W, Hg, Pb, Bi, and Th;

“Mehlich” (17 variables, 1333 training, and 571 validation samples), i.e., electrical conductivity (EC), exchangeable acidity (Ex. Ac.), exchangeable bases (Ex. Bas.), pH, phosphorus sorption index (PSI), and bioavailable forms of elements extracted with use of Mehlich 3 (M3) methodology, namely, M3 Al, M3 B, M3 Ca, M3 Cu, M3 Fe, M3 K, M3 Mg, M3 Mn, M3 Na, M3 P, M3 S, and M3 Zn.

For a description of the reference methods, in addition to standard operating procedures, see Vågen et al. [22]. The texture of the soil was determined with laser diffraction particle size analysis. For AfSIS reference samples, soil moisture release curves are determined using soil fines in pressure plate apparatus. High throughput total x-ray fluorescence spectroscopy (TXRF) was used in the analysis of the chemical composition (macro and microelements). Total and organic C and total N were determined with combustion method. Extractable Al, Ca, Mg, P, K, Na, S, Fe, Mn, Zn, Cu, B, Mo, H, and other bases were determined with use of ICP analysis of Mehlich 3 extracts. P sorption index was measured by single-point P addition.

**Figure 2:**
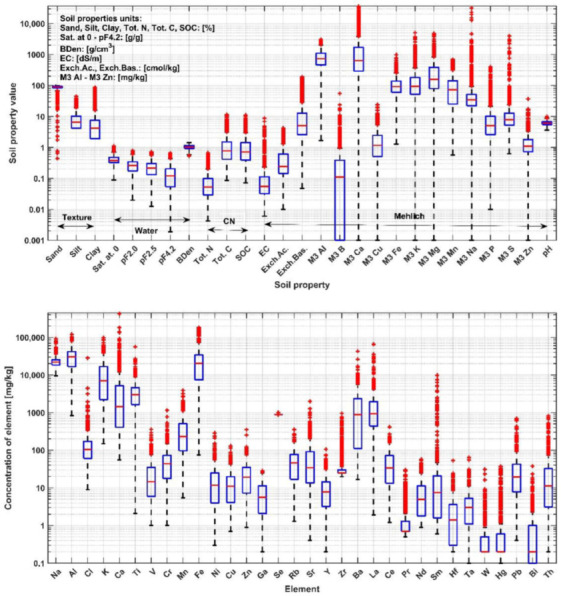
Box plots for modeled soil properties divided into separate data groups, i.e., Elements, Texture, Water, CN, and Mehlich. Blue boxes represent values between first and third quartile, red line represents median value and red crosses represent values outside the range of first quartile minus 1.5 times interquartile range and third quartile plus 1.5 times interquartile range.

Figure 2 and Appendix A (Table A1, Table A2, Table A3, Table A4 and Table A5) present selected statistics for all variables in these data groups. For each data group, prediction models were created for all soil properties included in the group.

### 2.2. Models

Three different groups of algorithms were used in this study: partial least squares regression (PLSR), one-dimensional convolutional neural network (1DCNN), and generalized regression neural network (GRNN).

The PLSR algorithm has been the most widely described and applied to prediction of the state of the environment from remotely sensed data. The deterministic PLSR algorithm [30,31] produces a linear model preceded by an orthogonalization operation of the input data taking into account their effect on the variance of the outputs. The experiments used a module available in the MATLAB package [32]. Depending on the number of model outputs, the number of PLS coefficients was set in range from 25 to 100. The single input vector consisted of 1749 reflectance values recorded for one soil sample.

A one-dimensional convolutional neural network [33] represents the so-called adaptive deep learning by processing the input data through successive layers of the algorithm. All conducted computational experiments used a network with two convolutional layers, counting 32 and 64 filters, or 64 and 128 filters with widths of three, six, and ten input features separated by MaxPooling layers. A fully connected layer consisted of 15 to 50 units. The number of outputs corresponded to the number of modeled properties. The spectra were standard normal variate (SNV)-transformed [34] and, like the outputs, were normalized to the numerical interval <0; 1> according to the methodology described in Gruszczyński and Gruszczyński [35]. The calculations associated with this algorithm were performed in the Python language environment using the Keras system and TensorFlow libraries [36,37].

**Figure 3:**
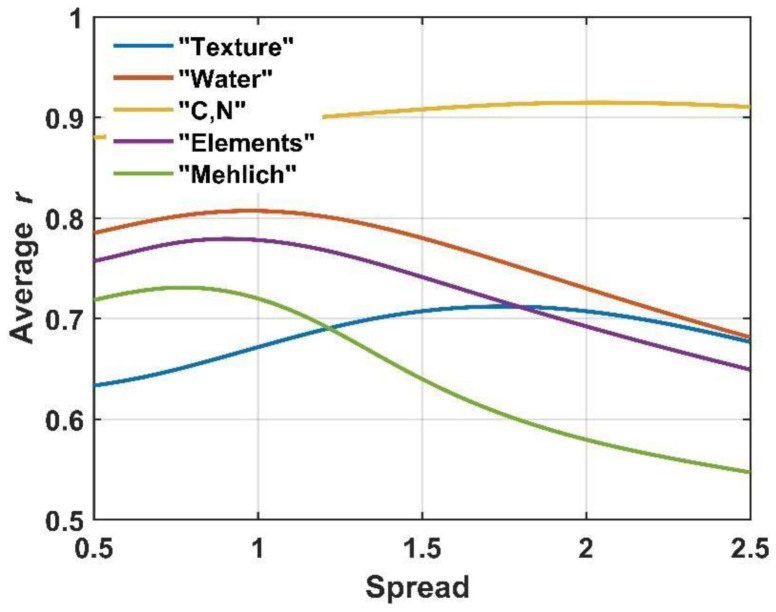
Dependence of the linear correlation coefficient between the reference and modeled values as a function of the spread value.

A generalized regression neural network is a memory-based local nonparametric linear and nonlinear regression algorithm [38]. The GRNN algorithm is susceptible to the curse of dimensionality; therefore, the spectral data from the AfSIS project were transformed into vectors of 50 components each using the PCA algorithm, considering all available spectra (18,250). In order to optimize the spread of RBF units, a modeling cycle was performed with its values between 0.5 and 2.5 (with a step of 0.05). Figure 3 shows the dependence of linear correlation coefficients of data and prediction as a function of spread. In the construction of models, the spread generating the highest average linear correlation coefficient of the prediction with the observed (r) data was used. Optimal spread values ranged from 0.75 for the “Mehlich” data group to 2.0 for variables C and N. Calculations associated with the GRNN model were performed in the MATLAB environment [32].

The evaluation criteria for each model were the following validation statistics: coefficient of determination (*R*^2^), root mean square error (*RMSE*), ratio of performance to interquartile distance (*RPIQ*), *bias*, standardized bias (*Sb*), and Lin’s concordance correlation coefficient (*LCCC*).

The coefficient of determination was calculated according to the equation:

(1) $$R^{2}=1-\frac{SSRv}{SSTv}$$

where *SSRv* is a sum of squares of differences of modeled value and its prediction in the validation set, and *SSTv* is a sum of squares of differences of modeled value and its mean in the validation set. The coefficient of determination is considered to be a numerical estimate of the degree to which the variance in a modeled variable is explained.

Root mean square error was calculated according to the equation:

(2) $$RMSE=\sqrt{\frac{SSRv}{n}}$$

where *n* is the size of the validation set.

Ratio of performance to interquartile distance was calculated according to the equation:

(3) $$RPIQ=\frac{IQR}{RMSE}$$

(4) $$IQR=Q_{3}-Q_{1}$$

where *IQR* is an interquartile distance, and $Q_{3}$ and $Q_{1}$ are third and first quartile of validation data.

The *bias* is calculated according to equation:

(5) $$bias=\frac{1}{n}\sum^{\;}\left(x_{i}-y_{i}\right)$$

where *x_i_* and *y_i_* are *i*th observed value and its prediction.

Standardized bias is calculated according to equation:

(6) $$Sb=\frac{bias}{IQR}$$

Lin’s concordance correlation coefficient *LCCC* [39] is calculated according to equation:

(7) $$LCCC=\frac{\left(2\cdot r\cdot s_{x}\cdot s_{y}\right)}{s_{x}^{2}+s_{y}^{2}+{\left(\underline{x}-\underline{y}\right)}^{2}}$$

where $s_{x}$, $s_{y}$ are standard deviations of observed values and their predictions, and $\underline{x}$ and $\underline{y}$ are the average of the observed values and their predictions. The *LCCC* value indicates the degree of agreement between the observed data and their prediction.

### 2.3. Hierarchical Summary of Prediction Positions

The relatively large number of modeled variables and the use of three different modeling algorithms justify the need to hierarchize the prediction accuracy of the variables in order to compare their practical utility. The hierarchization procedure was implemented using the technique for order of preference by similarity to ideal solution (TOPSIS) methodology [40]. This technique is used to rank objects described by multidimensional criteria through determination of their distance in multidimensional space from the potentially most favorable and least favorable variants. These variants are determined by identifying, for each criterion, the highest and lowest rated values. As a result, two (not necessarily existing in the dataset) variants are created with the best and the worst properties. The distance, in the multidimensional space, from both of them is a measure of the quality of prediction of the variable, according to the formula:

(8) $$TIndex=\frac{DistWORST}{DistWORST+DistBEST}$$

where *TIndex* is a ranking coefficient, *DistWORST* is a euclidean distance in multidimensional space from the worst variant, and *DistBEST* is a euclidean distance in multidimensional space from the most favorable variant.

The criteria used in TOPSIS method to characterize the prediction quality should be independent of the values scale. Thus, *R*^2^, *RPIQ*, *Sb*, and *LCCC* statistics scaled to the interval <0; 1> were used. The prediction accuracy of the model is highly dependent on the data number, distribution, and range of variability. For this reason, the hierarchization performed is of relative importance, depending on the type and homogeneity of the data.

### 2.4. Importance of Spectral Bands to Soil Property Estimation

The multidimensionality of spectral data is a significant obstacle in some modeling algorithms. The reduction of the dimensionality of the input data is a component of linear models (PCA, PLS). It is always associated with determining the parts of the spectrum that are important for obtaining a good estimation of the sought relationship between the spectral response and soil properties.

In PLSR models, the significance of individual parts of the spectrum for the estimation of a soil variable can be determined by calculating the indicator referred to as (VIP scores) variable importance in projection [30,32]. VIP scores, as a vector of numerical values with a length equal to the number of spectral registration points, can be calculated a posteriori. The sum of squares of the VIP scores for the spectrum is equal to the number of spectrum components. VIP scores less than 1.0 indicate a lower than average importance of a particular input value. Components with VIP scores greater than 1.0 are important for modeling. To assess the importance of spectral bands, calculations of VIP scores for 11 selected soil features (SOC, total N, Ca, Al, Fe, Cr, Cu, Pb, Zn, and Hg contents) were performed during the creation of PLSR models for these properties.

An independent analysis of the significance of spectral bands was performed with the neighborhood components analysis (NCA) algorithm. This algorithm, not related to a specific modeling method, is used to select the number of input variables a priori, especially in the case of a limited number of data [32,41,42,43]. In order to compare the results of this approach with VIP scores, calculations were performed on the same dataset.

## 3. Results

### 3.1. Results of Modeling of Texture

The search for an effective model to estimate soil texture is a frequent topic in soil science research [44]. The estimates of the content of the different fractions vary in terms of *RMSE* values (Table 1). Validation statistics for all algorithm groups analyzed are relatively weak $\left(R^{2}<0.75\right)$ and (RPIQ<2.0); *R*^2^, *RPIQ*, and *LCCC* signal significant agreement between predictions and observations, but *RMSE* is relatively high. In our study, the individual models unanimously indicate that sand has the largest prediction error, while silt and clay have much smaller errors. This is an indirect indication that when soil texture needs to be determined, it is safest to determine it using these two variables. However, it should be assumed that this result is specific to the AfSiS dataset and it results from a much larger concentration spread of Sand compared to the range of silt and clay. With similar $R^{2}$ values, the RMSEs for silt and clay fractions are significantly smaller. Moreover, the distribution of sand content is characterized by negative asymmetry.

### 3.2. Results of Modeling of Properties from “Water” Group

The complicated and lengthy process of laboratory determination of water characteristics of soils using sand box and ceramic plates justifies the search for alternative means of estimating them. The validation statistics of the prediction models for the “Water” group indicate that the models based on MIR provide a reasonably good basis for estimation of these quantities (Table 2). *RPIQ*s exceeding 2.0 signal a relatively good approximation of all relevant properties. Saturation at 0 and pF2.0 were estimated with the least error by the GRNN model; soil moisture at pF2.5 and pF4.2, by the 1DCNN model; while the volumetric density estimate with the highest accuracy is provided by the PLSR model.

### 3.3. Results of Modeling of C and N Contents

**Figure 4:**
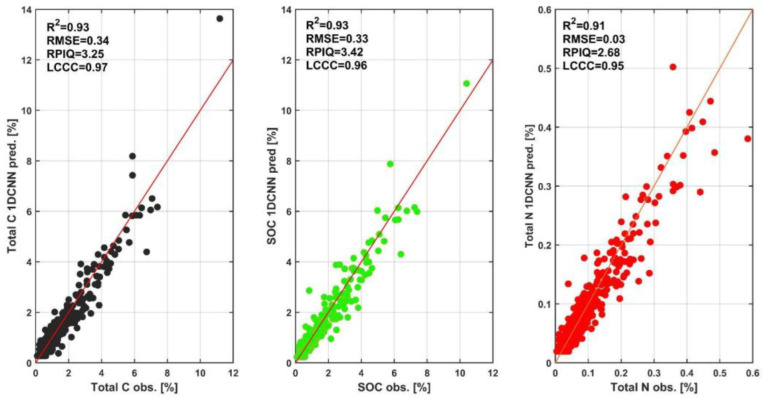
Scatter plots of C and N content prediction validation data using the 1DCNN model. When the points are on the red line it is an indication of perfect prediction, deviations from red line indicate prediction errors.

C and N concentrations in soils are the soil properties most commonly modeled, based on NIR and MIR spectral response [2,3]. This is due to the important role of both elements in shaping other soil properties, the role of N as a nutrient, and the importance of C sequestration in soils under conditions of increasing CO_2_ content in ambient air. Validation statistics (Table 3, Figure 4) signal that, in the AfSIS dataset, the concentration of total C and N, as well as SOC, can be determined with relatively high accuracy, even when the compound content of both elements in soils is low. *RMSE* estimates of 0.33% for C and 0.03% for N provide good approximations of the true values. Validation results indicate that (among those tested) the best model for estimating C and N is 1DCNN. This provides reason to believe that the relationship between the spectrum and SOC concentration (and associated N) is nonlinear, as indicated by the weaker result of the PLSR model. The result of the GRNN model is also relatively weak.

### 3.4. Results of Modeling of Properties from “Elements” Group

Table 4 contains validation statistics for the prediction models of the 32 elements. The validation results vary widely, although there are several elements whose concentrations can be estimated with relatively high accuracy. The *RPIQ* value indicates that a small relative error characterizes the prediction of Fe, Al, Ga, and Rb (*RPIQ* > 2.0). However, analyzing the *RMSE* level shows that, despite low values of *RPIQ* or *R*^2^, the content of other macroelements can also be predicted with sufficient accuracy, taking into account the scale of data variability (data spread). Comparison of the validation results from the three models indicates that the GRNN model dominates in terms of prediction quality. There are six exceptions to this rule: Na, K, Ca, and Se, for which better prediction results were obtained with the PLSR method; and Se and Bi, for which a slightly better result was obtained with the 1DCNN model.

### 3.5. Results of Modeling of Properties from “Mehlich” Group

Table 5 shows the prediction statistics of chemical properties of soils from the “Mehlich” group. Apart from Al content and pH value, the *RPIQ* of other characteristics does not exceed a value of 2.0. A relatively good validation result is shown by the model of Ca and K content (PLSR), and Mg content (1DCNN).

### 3.6. Relative Prediction Positions

Table 6 contains hierarchical summaries of the relative prediction positions of soil variables using the TOPSIS method considering the *R*^2^, *RPIQ*, *LCCC*, and *Sb* criteria. A high position in the hierarchy indicates a prediction result more consistent with the observations. Hierarchization takes into account all validation results. A higher Tindex value for the same predicted element indicates an algorithm that more accurately approximates the variables. Despite some differences in the position of individual variables, a relatively narrow group of data can be predicted with the greatest precision: SOC, Al and M3 Al, Fe, K, pH, and total N. Ranks of prediction of soil texture elements and water properties are relatively low. However, this highly formalized hierarchical procedure does not invalidate the usefulness of lower-ranked models, which should be further evaluated. This applies, for example, to the prediction of Ca or pF, especially at higher values of these variables.

Spectral response is used for the estimation of soil properties in digital mapping of the environment as well as in the management of field operations. The use of spectral analysis in laboratory conditions should be included in a similar category as it allows rapid, inexpensive, and approximate estimation of some soil properties in the whole soil profile [45,46]. An important problem is that of identifying the optimal modeling algorithm, as well as the limiting prediction precision, taking into account the minimum documentation requirements.

### 3.7. Impact of Relationships between Properties

It can be assumed that in the field of spectral analysis used for quantitative estimation of soil properties, there are three categories of variables: those that affect the spectral response directly to the extent that they can be quantitatively identified; those that are correlated with properties that affect the spectral response and thus can be quantitatively estimated; and those that do not affect the spectral response and are not correlated with properties that affect the spectral response. Results of previous studies indicate that, for soils, the first category includes carbon organic compounds [47,48], when using both NIR and MIR spectra. For other features, the possibility of belonging to each category has to be evaluated experimentally.

**Figure 5:**
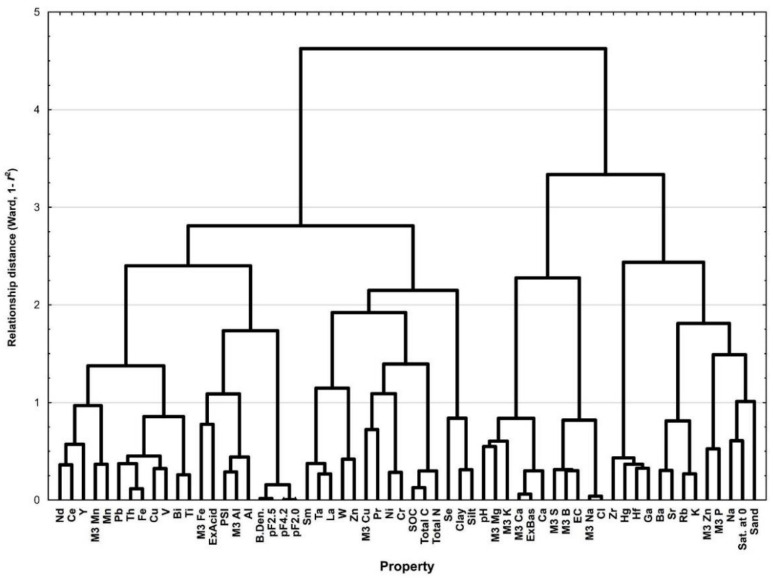
Dendrogram of associations between variables obtained by Ward’s agglomeration method.

The linkages between soil properties that enable or interfere with the quantitative identification of some components can be traced by confronting the statistics of the prediction results with the cluster system generated by the variable agglomeration algorithm. Figure 5 shows the dendrogram of associations between soil properties obtained by Ward’s agglomeration procedure using the 1–*r^2^* metric [49,50]. The algorithm groups statistically related variables using a metric that expresses the similarity or dissimilarity between variables. The value of the association distance indicates the degree of similarity of the variables in the dataset. A cluster composed of mutually correlated variables Ti, Bi, V, Cu, Fe, Th, Pb, and Mn in models using MIR is characterized by coefficients of determination of, respectively: 0.67, 0.35, 0.63, 0.59, 0.84, 0.76, 0.64, 0.62. The highest *R*^2^ value of the Fe prediction leader gives reason to believe that the variability of its content reflects to some extent the variability of the other elements of the cluster, supporting their prediction. The cluster related to Fe is in turn related to the cluster including M3 Cu, Pr, Ni, Cr, M3 Fe, Ex. Acid., PSI, M3 Al, and Al (*R*^2^: 0.50, 0.33, 0.66, 0.68, 0.49, 0.39, 0.78, 0.86, 0.79, respectively). There is also a cluster of variables related to soil pH, including pH, M3 Mg, M3 K, M3 Ca, Ex. Bas., and Ca. A characteristic group is formed by variables shaping water properties including: bulk density, pF2.0, pF2.5, pF4.2, SOC, total C, and total N. In this case, one might suspect that the supporting factor for quantitative identification in this group is total C, or SOC.

**Figure 6:**
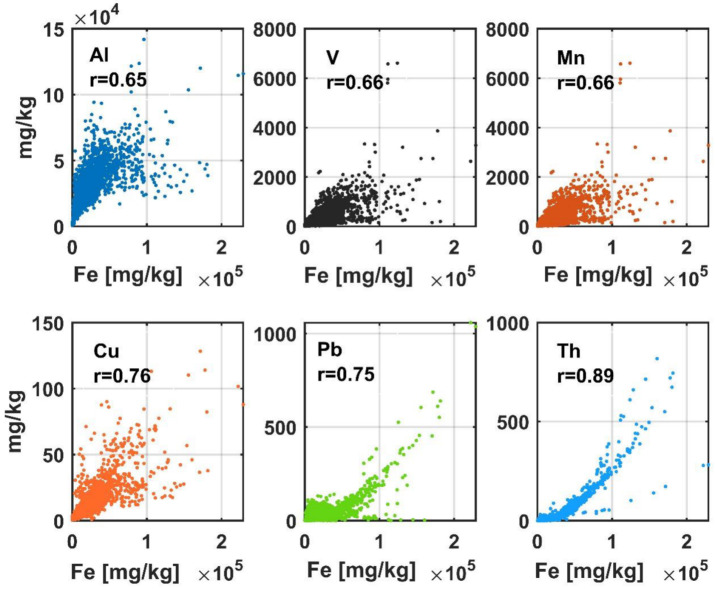
Scatter plots of the content of selected elements as a function of Fe content.

The graphs in Figure 6 illustrate the statistical relationships between Fe content and the concentration of other metals (Al, V, Mn, Cu, Pb, and Th). It can be assumed that the model of these elements’ content is to some extent related to the content of Fe, which is well quantified by the models. It cannot be excluded that the chain of connections between particular properties is more complex and may lead to correct estimation of features poorly influencing the spectral response or not influencing it at all.

**Figure 7:**
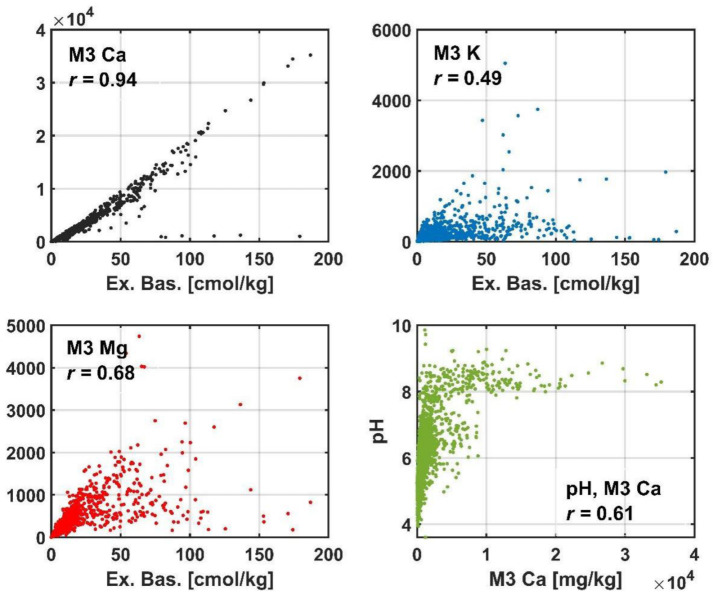
Scatter plots of soil variables significantly correlated with the exchangeable bases and Ca content.

Similar relationships exist between the exchangeable bases and the elements included in them (Figure 7). The exchangeable bases value is shaped by Ca to the largest extent, although the association with K and Mg is also significant. Calcium content, fairly well identified through the MIR spectral response, is nonlinearly related to pH. It is difficult to indicate which of these elements is the primary basis for correct modeling of soil sorption elements.

### 3.8. Importance of Spectral Bands in Prediction of Selected Properties

**Figure 8:**
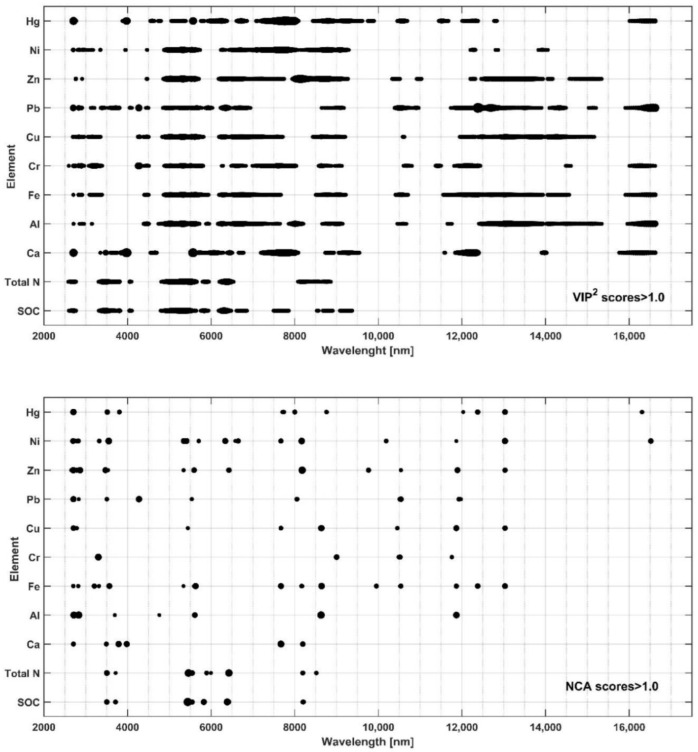
Distribution diagrams of squares of VIP scores and NCA scores for selected soil properties. The diagrams include index values exceeding 1.0. The diameters of points are proportional to the squares of VIP scores and NCA scores.

Figure 8 shows the diagrams of squares of VIP scores and NCA score values for 11 soil characteristics, including the concentration of heavy metals. The diagrams reflect the properties of the PLSR and NCA algorithms. In the case of PLSR, most orthogonalized inputs are used in the construction of the model. In the selection, in the NCA procedure, only spectral sections with appropriately high NCA scores are considered. In the analyzed cases, the number of values indicated by this procedure ranged from 12 to 54.

It can be seen that SOC and total N is determined by taking the same spectral segments into account. It can also be concluded that the concentration of Al, and especially Fe, is related to sections of the spectrum of heavy metals.

## 4. Discussion

Most soil data, for classification purposes or for making economic decisions, are interpreted in a semi-fuzzy way, or at least a tendency is observed leading to a blurring of the boundaries between classification criteria. The evaluation of modeling results of many soil characteristics must allow for a certain level of acceptable errors. In this context, the estimation of soil texture components, such as silt and clay contents, using the models described in this paper, can be considered as sufficiently precise. Similarly, the correctness of the models of water properties of soils, which are extremely time-consuming to determine in the laboratory using the classical approaches, should be assessed.

The modeling of SOC concentration based on NIR and MIR signatures is understandably of the greatest interest to researchers [2,3,4,51]. It can be assumed that, as an important component of soil organic compounds, SOC and total N content can be estimated with satisfactory accuracy. In terms of total element prediction, only a few elements can be estimated with reasonable accuracy: Al, Fe, Ca, and K, along with several elements usually found in soils in low concentrations, namely Se, Rb, Ga, Zr, La, and Th. The contents of Cr, Ti, Mn, Ni, and Pb are estimated with slightly lower accuracy.

In modeling the “Mehlich” data group (17 variables), the 1DCNN (10 variables with the best prediction statistics) and GRNN (7 variables with the best prediction statistics) algorithms performed best. The PLSR model was inferior to the other two in every case. Particularly good prediction scores apply to: M3 Al, M3 B, M3 Mg, and pH (1DCNN model); and to total exchangeable bases, M3 Ca, M3 K, M3 Na, and PSI (GRNN model).

The results presented indicate that, among the examined models, there is no single optimal one that would provide the best predictions for all variables. Each of the models shows advantages in predicting a certain group of variables and at the same time is less useful in predicting others. This has certainly been influenced by the heterogeneity of data from many different countries, which is evident through the very large spread of variable values [24]. Compared to results with less dispersed distributions, more than 100 variables across the United States from the Kellogg Soil Survey Laboratory [8] show, in many cases, similar values of determination coefficients, but are associated, in the case of AfSIS data, with a much larger *RMSE* values. Comparisons between the two sets of results are difficult, mainly due to the smaller spread—often by several times—of the Kellogg Laboratory data relative to AfSIS, and also due to the slightly wider spectral range they used. Data from the USA were also used by Wijewardane et al. [9], who indicated satisfactory results for prediction of C, N, CEC, pH, and clay content, while poor results were produced for extractable forms of phosphorus and potassium. In this case, the results (*RMSE*) are similar to those obtained from the AfSIS data, presumably also due to less restrictive data selection conditions that did not limit the spread of the data. AfSIS observational data have been used in very advanced digital mapping attempts. Hengl et al. [25] presented a modeling methodology to generate a map of soil properties, among other things, from data collected in the AfSIS project. They used an ensemble of machine learning regression models whose inputs were topographic, hydrological, meteorological, and vegetation data, as well as spectral reflectance images collected by the Sentinel satellites (Copernicus program). Nineteen variables were modeled, mainly Mehlich 3 extractable components, pH, texture, CEC, C, and N contents. Results of modeling based on spectra recorded from the satellite ceiling were slightly worse than those obtained by proximal sensing. The disadvantage of this approach is the limitation of quantitative identification of variables to the surface layer. The indisputable advantage, however, is the possibility of relatively precise representation of many soil properties with good spatial resolution over a very large area.

If we arbitrarily take $\left(R^{2}\geq 0.75\right)$ and (RPIQ≥2.0) as the criteria of correct prediction, then for PLSR models they are fulfilled for 10 of the studied soil properties. For nine variables, these criteria are met when 1DCNN is used, while among predictions using the GRNN model, the adopted criteria are met for seven variables. For six variables, the assumed criteria are met by all tested algorithms. These variables are SOC, total C, Al, Fe, M3 Al, and pH.

The decision to use a model based on spectral analysis instead of the reference method is inevitably subjective. Using validation statistics, it is possible to estimate the risk of error based, for example, on *R*^2^ and *RMSE* values. Acceptance or rejection of the model as an approximation for classification or mapping of soils is determined by the mapping scale (spatial resolution) and the way in which the variability of soils is reflected—either as a continuous image or discrete representation with information about the mean value of properties and the range of their variability.

Assuming a symmetrical distribution of deviations from observed values, properties such as sand, clay; total C, N, and SOC contents; and bulk density can be estimated with sufficient precision for large-scale soil mapping. In addition, the Ca content error of 0.5% relative to sample weight is small enough even for assessing the cultivation needs of soils, although the K content (error of 0.7%) is estimated with relatively low accuracy. Fe compounds rarely show signs of deficiency; therefore, an error of 1% is not significant in practice. Similarly, Al concentration itself does not pose a significant problem; it only becomes important once the soil pH is below 5.0, when the presence of aluminum compounds is a threat to crops. The accuracy of estimates of other variables must be considered depending on the purpose and scale of the documentation required. For example, in the context of investigating threats to soils with high metal contents, even estimates of relatively low accuracy may be sufficient to determine the extent of transformation.

**Figure 9:**
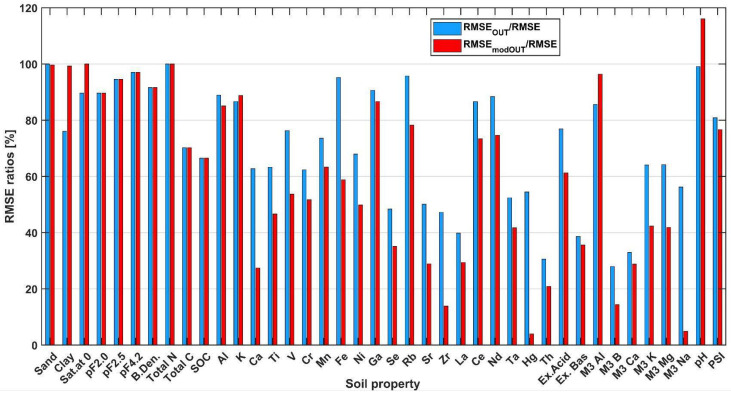
Example *RMSE* ratios.

For large soil datasets, especially those from diverse geological settings, a significant asymmetry in the distribution of soil characteristics, usually right-handed, is characteristic. The aforementioned data from the US territory analyzed by Ng et al. [8] possess such a characteristic. Towett et al. [24], characterizing the chemical properties of the AfSIS data, noted the strong skewness of the distributions of many soil variables. Relative to the distribution of US data, the spread of AfSIS observations is, in most cases, much larger (in extreme cases, several tens of times). This has a significant impact on the level of prediction errors, especially at low values of the variables. In such circumstances, one of the solutions used is to exclude extremely high values (outliers) from the dataset. Such an operation, in the context of the real occurrence of high values of variables, is controversial. Figure 9 illustrates, based on the analyzed data, the relationship between the prediction error of the model with the most favorable characteristics (*RMSE*) and the prediction errors after removing the values considered as outliers, i.e., exceeding the assumed cut-off value (HL):

$$HL=Q_{3}+1.5\cdot IQR$$

The graph shows two versions of this approach: the *RMSE* ratio after removing extreme values from the validation data after prediction with a model derived from the entire data (*RMSE*_OUT_); and the *RMSE* ratio after removing extreme values from the training and validation data before training the model (*RMSE*_modOUT_).

A significant variation in the ratios of *RMSE* values can be seen. As a rule, the error after removing outliers before training is smaller than the *RMSE* for the full data and the *RMSE* after removing outliers from the validation data after training on the full data. The exceptions are clay, Sat. at 0, K, M3 Al, and pH, for which limiting the range of variables in the learning set worsened the validation results. For sand and total N content, the exclusion of extreme feature values did not significantly affect the validation error.

The largest decreases in *RMSE* are for M3 Na, M3 K, M3 B, Hg, and Zr. Other *RMSEs* are closer to the variant with removal of outliers after training on the whole data. The problem of outliers for prediction of soil property values is debatable. The increase in accuracy due to truncation of large data values limits the applicability of the model, especially for memory-based models. In extreme cases, the construction of two different models should be considered [21]: one for data close to the modal and mean value, and a separate one for very high values of the variables. However, for models built for high property values, the problem will be the small number of learning examples.

## 5. Conclusions

Each of the models examined possesses a specific suitability for prediction of different soil variables. It should be assumed that differences in the correctness of estimation of particular variables depend on the type of relationship between the variable and the spectral response. This means that, due to the specificity of the relationship between the MIR spectrum and soil properties, it is not possible to indicate a universal optimal prediction model for all soil variables. Because of the wide range of the variable distributions, this result should be treated as specific for particular geological, morphological, and climatic conditions.

The best prediction results are provided by all regression models for total C, SOC, total Fe, Al, M3 Al, and pH. The clustering and modeling results indicate that many other soil properties do not directly affect the MIR spectral response, but there are correlations of features that can support such a prediction with limited accuracy.

Asymmetry in the distribution of the modeled variables makes it difficult to build a valid regression model. Removing outliers from the training set results in a significant reduction of the prediction error in many cases. However, this operation limits the universality of the model to a range relatively close to the median. Consideration of building two different models for different ranges of data variability is conditioned by the disproportion of the number of examples close to the median and outliers.

## Figures and Tables

**Table 1:** Summary of validation statistics for models in the “Texture” group. The best prediction result for particular variables is marked in bold.

Property		PLSR					1DCNN					GRNN				
		*R* ^2^	*RMSE*	*RPIQ*	*LCCC*	*Sb*	*R* ^2^	*RMSE*	*RPIQ*	*LCCC*	*Sb*	*R* ^2^	*RMSE*	*RPIQ*	*LCCC*	*Sb*
Sand	[%]	**0.62**	**8.83**	**1.08**	**0.79**	**0.05**	0.60	9.02	1.05	0.73	−0.02	0.54	9.65	0.98	0.72	−0.01
Silt	[%]	**0.54**	**4.04**	**1.41**	**0.73**	**0.00**	0.37	4.71	1.21	0.59	0.31	0.32	4.90	1.16	0.56	0.03
Clay	[%]	0.64	5.72	0.93	0.80	−0.04	0.64	5.74	0.93	0.77	0.28	**0.66**	**5.54**	**0.97**	**0.79**	**0.03**

**Table 2:** Summary of model validation statistics for the “Water” group. The best prediction result for particular variables is marked in bold.

Property		PLSR					1DCNN					GRNN				
		*R* ^2^	*RMSE*	*RPIQ*	*LCCC*	*Sb*	*R* ^2^	*RMSE*	*RPIQ*	*LCCC*	*Sb*	*R* ^2^	*RMSE*	*RPIQ*	*LCCC*	*Sb*
Sat. at 0	[g/g]	0.61	0.09	1.64	0.77	0.04	0.61	0.09	1.66	0.77	−0.05	**0.70**	**0.08**	**1.89**	**0.82**	**0.07**
pF2.0	[g/g]	0.55	0.08	2.05	0.73	0.05	0.60	0.08	2.17	0.76	0.00	**0.62**	**0.08**	**2.24**	**0.77**	**0.05**
pF2.5	[g/g]	0.56	0.08	2.20	0.74	0.05	**0.62**	**0.07**	**2.38**	**0.77**	**−0.01**	0.62	0.07	2.38	0.77	0.05
pF4.2	[g/g]	0.49	0.08	1.94	0.67	0.06	**0.59**	**0.07**	**2.15**	**0.73**	**0.01**	0.56	0.07	2.10	0.72	0.06
B. Den.	[g/cm^3^]	**0.76**	**0.08**	**2.96**	**0.86**	**0.01**	0.74	0.08	2.83	0.85	0.03	0.73	0.08	2.76	0.84	0.00

**Table 3:** Summary of model validation statistics for C and N concentration in soils. The best prediction result for particular variables is marked in bold.

Property		PLSR					1DCNN					GRNN				
		*R* ^2^	*RMSE*	*RPIQ*	*LCCC*	*Sb*	*R* ^2^	*RMSE*	*RPIQ*	*LCCC*	*Sb*	*R* ^2^	*RMSE*	*RPIQ*	*LCCC*	*Sb*
Total N	[%]	0.84	0.03	2.03	0.91	0.01	**0.91**	**0.03**	**2.68**	**0.95**	**0.00**	0.80	0.04	1.85	0.88	−0.01
Total C	[%]	0.92	0.38	2.95	0.96	0.00	**0.93**	**0.34**	**3.25**	**0.97**	**0.05**	0.87	0.47	2.37	0.93	−0.01
SOC	[%]	0.91	0.37	3.02	0.96	0.00	**0.93**	**0.33**	**3.42**	**0.96**	**0.05**	0.86	0.47	2.39	0.92	−0.02

**Table 4:** Summary of model validation statistics for the “Elements” group. The best prediction result for particular variables is marked in bold.

Property		PLSR					1DCNN					GRNN				
		*R* ^2^	*RMSE*	*RPIQ*	*LCCC*	*Sb*	*R* ^2^	*RMSE*	*RPIQ*	*LCCC*	*Sb*	*R* ^2^	*RMSE*	*RPIQ*	*LCCC*	*Sb*
Na	[mg/kg]	**0.46**	**6452.1**	**1.09**	**0.64**	0.04	0.36	7029.8	1.00	0.54	0.13	0.44	6553.6	1.07	0.62	−0.01
Al	[mg/kg]	0.77	8714.3	2.87	0.87	−0.02	0.76	8854.0	2.83	0.86	−0.01	**0.79**	**8377.4**	**2.98**	**0.88**	**0.00**
Cl	[mg/kg]	0.00	1592.5	0.07	0.43	−0.79	0.00	1199.5	0.09	0.06	0.44	**0.30**	**1013.5**	**0.11**	**0.44**	**−0.37**
K	[mg/kg]	**0.83**	**4990.3**	**2.89**	**0.91**	−0.03	0.79	5437.6	2.66	0.89	0.07	0.72	6299.1	2.29	0.84	−0.06
Ca	[mg/kg]	**0.95**	**5148**	**0.93**	**0.98**	−0.05	0.61	14,979	0.32	0.75	−0.36	0.94	5767	0.83	0.97	0.04
Ti	[mg/kg]	0.41	3203.3	0.93	0.69	−0.05	0.36	3342.7	0.89	0.59	0.15	**0.67**	**2422.0**	**1.22**	**0.81**	**−0.04**
V	[mg/kg]	0.51	27.98	1.04	0.72	−0.10	0.49	28.59	1.01	0.64	−0.02	**0.63**	**24.12**	**1.20**	**0.78**	**−0.08**
Cr	[mg/kg]	0.40	64.72	0.85	0.60	−0.10	0.30	70.07	0.79	0.47	0.05	**0.68**	**47.08**	**1.17**	**0.81**	**−0.02**
Mn	[mg/kg]	0.44	336.76	1.21	0.70	−0.02	0.54	304.55	1.34	0.71	0.07	**0.62**	**278.77**	**1.46**	**0.76**	**−0.03**
Fe	[mg/kg]	0.79	12,398	2.13	0.89	−0.01	0.79	12,624	2.10	0.86	0.07	**0.86**	**10,274**	**2.57**	**0.92**	**−0.01**
Ni	[mg/kg]	0.47	19.70	1.05	0.70	−0.11	0.54	18.30	1.13	0.71	−0.06	**0.66**	**15.75**	**1.31**	**0.79**	**−0.01**
Cu	[mg/kg]	0.55	10.04	1.56	0.72	0.01	0.52	10.38	1.50	0.72	−0.01	**0.59**	**9.64**	**1.62**	**0.74**	**0.00**
Zn	[mg/kg]	0.41	21.48	1.27	0.63	−0.02	0.37	22.13	1.24	0.64	−0.09	**0.46**	**20.57**	**1.33**	**0.66**	**−0.01**
Ga	[mg/kg]	0.68	3.36	2.73	0.82	−0.04	0.63	3.59	2.56	0.79	−0.12	**0.73**	**3.07**	**2.99**	**0.84**	**0.00**
Se	[mg/kg]	0.71	9.00	0.36	0.82	0.24	0.59	10.75	0.30	0.72	−0.96	**0.81**	**7.37**	**0.43**	**0.89**	**0.11**
Rb	[mg/kg]	0.71	25.31	2.38	0.84	−0.02	0.65	28.00	2.15	0.78	0.11	**0.72**	**25.07**	**2.40**	**0.83**	**−0.03**
Sr	[mg/kg]	0.42	118.85	0.65	0.68	0.01	**0.63**	**94.37**	**0.82**	**0.76**	**0.10**	0.49	111.22	0.69	0.63	0.02
Y	[mg/kg]	0.42	8.69	1.30	0.63	−0.05	0.40	8.83	1.27	0.59	−0.05	**0.48**	**8.23**	**1.37**	**0.65**	**−0.02**
Zr	[mg/kg]	0.47	83.82	0.06	0.71	−1.86	0.58	74.20	0.07	0.69	0.93	**0.77**	**55.06**	**0.09**	**0.86**	**0.22**
Ba	[mg/kg]	0.39	2331.2	0.96	0.64	−0.05	0.26	2560.7	0.87	0.42	0.03	**0.44**	**2223.5**	**1.00**	**0.62**	**0.02**
La	[mg/kg]	0.48	3190.5	0.47	0.62	0.12	0.39	3447.5	0.44	0.52	0.34	**0.82**	**1874.5**	**0.80**	**0.89**	**0.06**
Ce	[mg/kg]	0.36	38.65	1.51	0.67	−0.04	0.41	37.10	1.57	0.67	0.05	**0.56**	**31.97**	**1.83**	**0.75**	**−0.06**
Pr	[mg/kg]	0.26	2.92	0.21	0.42	0.44	0.15	3.13	0.19	0.22	0.25	**0.33**	**2.78**	**0.22**	**0.47**	**0.60**
Nd	[mg/kg]	0.42	6.69	1.40	0.68	−0.04	0.41	6.73	1.40	0.61	−0.05	**0.62**	**5.42**	**1.73**	**0.78**	**−0.06**
Sm	[mg/kg]	0.34	600.16	0.03	0.46	1.75	0.17	671.35	0.03	0.24	4.85	**0.58**	**477.83**	**0.04**	**0.72**	**0.65**
Hf	[mg/kg]	0.14	4.04	0.82	0.54	−0.16	0.05	4.26	0.77	0.29	0.27	**0.40**	**3.39**	**0.97**	**0.59**	**−0.08**
Ta	[mg/kg]	0.54	4.50	0.91	0.70	0.02	0.37	5.31	0.77	0.48	0.03	**0.73**	**3.43**	**1.19**	**0.84**	**0.01**
W	[mg/kg]	0.37	1.78	0.17	0.57	0.07	0.18	2.03	0.15	0.31	0.43	**0.41**	**1.72**	**0.17**	**0.64**	**−0.11**
Hg	[mg/kg]	0.52	3.36	0.12	0.76	−1.12	0.46	3.57	0.11	0.68	−2.07	**0.65**	**2.85**	**0.14**	**0.85**	**−0.78**
Pb	[mg/kg]	0.34	54.29	0.64	0.59	−0.02	0.39	52.50	0.66	0.53	0.22	**0.70**	**36.57**	**0.94**	**0.81**	**0.00**
Bi	[mg/kg]	0.02	3.02	0.30	0.62	−0.55	0.35	2.47	0.36	0.66	0.19	**0.56**	**2.01**	**0.45**	**0.76**	**−0.25**
Th	[mg/kg]	0.65	54.15	0.54	0.77	0.04	0.63	56.12	0.52	0.71	0.39	**0.77**	**43.79**	**0.67**	**0.86**	**0.06**

**Table 5:** Summary of model validation statistics for the “Mehlich” group. The best prediction result for particular variables is marked in bold.

Property		PLSR					1DCNN					GRNN				
		*R* ^2^	*RMSE*	*RPIQ*	*LCCC*	*Sb*	*R* ^2^	*RMSE*	*RPIQ*	*LCCC*	*Sb*	*R* ^2^	*RMSE*	*RPIQ*	*LCCC*	*Sb*
EC	[dS/m]	0.19	0.28	0.29	0.47	−0.05	**0.38**	**0.25**	**0.34**	**0.64**	**−0.18**	0.31	0.26	0.32	0.50	0.16
Ex. Ac.	[cmol/kg]	0.40	0.45	1.15	0.58	0.02	**0.55**	**0.39**	**1.33**	**0.73**	**0.02**	0.33	0.48	1.09	0.57	0.07
Ex. Bas.	[cmol/kg]	0.82	9.99	1.01	0.91	0.01	0.88	8.05	1.26	0.94	0.05	**0.90**	**7.50**	**1.35**	**0.95**	**0.02**
M3 Al	[mg/kg]	0.84	192.32	3.43	0.92	0.01	**0.86**	**181.99**	**3.63**	**0.92**	**0.01**	0.85	189.46	3.48	0.92	0.00
M3 B	[mg/kg]	0.39	1.33	0.26	0.55	0.11	**0.70**	**0.93**	**0.37**	**0.80**	**0.23**	0.37	1.35	0.26	0.51	0.21
M3 Ca	[mg/kg]	0.82	1752.3	0.76	0.90	0.02	0.82	1741.9	0.76	0.90	0.04	**0.89**	**1394.7**	**0.96**	**0.94**	**0.02**
M3Cu	[mg/kg]	0.19	1.90	0.98	0.38	−0.03	0.33	1.73	1.07	0.53	−0.04	**0.50**	**1.49**	**1.25**	**0.70**	**−0.04**
M3 Fe	[mg/kg]	0.42	70.28	1.26	0.57	0.06	**0.49**	**65.81**	**1.34**	**0.63**	**0.04**	0.39	72.11	1.22	0.56	0.07
M3 K	[mg/kg]	0.18	227.55	0.57	0.55	−0.13	0.40	194.01	0.66	0.56	0.18	**0.65**	**147.94**	**0.87**	**0.80**	**−0.04**
M3 Mg	[mg/kg]	0.68	234.54	1.28	0.82	−0.03	**0.74**	**209.04**	**1.44**	**0.86**	**0.02**	0.68	232.25	1.29	0.81	0.01
M3 Mn	[mg/kg]	0.47	75.63	1.54	0.64	0.02	**0.58**	**67.78**	**1.72**	**0.73**	**0.04**	0.51	73.30	1.59	0.72	−0.04
M3 Na	[mg/kg]	0.00	1003.1	0.03	0.32	0.88	0.11	942.55	0.03	0.47	1.14	**0.89**	**325.35**	**0.09**	**0.83**	**0.85**
M3 P	[mg/kg]	0.02	28.66	0.25	0.15	−0.15	0.25	**25.06**	**0.28**	**0.39**	**0.35**	0.19	25.99	0.27	0.38	0.14
M3 S	[mg/kg]	0.12	199.34	0.04	0.20	1.06	0.28	180.35	0.05	0.50	1.23	**0.49**	**151.66**	**0.05**	**0.44**	**1.62**
M3 Zn	[mg/kg]	0.13	1.50	0.64	0.30	−0.15	**0.22**	**1.42**	**0.68**	**0.31**	**0.08**	0.11	1.52	0.64	0.34	−0.02
pH		0.81	0.48	2.80	0.90	−0.01	**0.84**	**0.43**	**3.07**	**0.91**	**−0.03**	0.77	0.52	2.56	0.87	0.00
PSI		0.75	45.09	1.96	0.85	0.02	0.72	47.35	1.86	0.83	0.14	**0.78**	**41.79**	**2.11**	**0.88**	**0.04**

**Table 6:** Hierarchical summary of relative predictive position of soil variables.

Rank	PLSR		1DCNN		GRNN	
	Property	Tindex	Property	Tindex	Property	Tindex
1	M3 Al	0.839	SOC	0.853	M3 Al	0.843
2	SOC	0.833	M3 Al	0.847	Al	0.800
3	Total C	0.831	Total C	0.846	Ga	0.780
4	K	0.809	pH	0.824	Fe	0.777
5	pH	0.796	Total N	0.808	Total C	0.776
6	B. Den.	0.790	Al	0.782	SOC	0.776
7	Al	0.789	K	0.777	B. Den.	0.762
8	Ga	0.748	B. Den.	0.772	pH	0.760
9	Total N	0.737	Fe	0.725	Rb	0.733
10	Fe	0.736	Ga	0.718	PSI	0.729
11	Rb	0.732	pF2.5	0.692	K	0.728
12	PSI	0.704	PSI	0.683	Total N	0.713
13	Ca	0.666	Rb	0.680	pF2.5	0.690
14	pF2.5	0.652	Ex. Bas.	0.678	Ex. Bas.	0.689
15	Ex. Bas.	0.646	pF2.0	0.667	Sat. at 0	0.682
16	pF2.0	0.638	M3 Mg	0.660	pF2.0	0.680
17	Sat. at 0	0.630	pF4.2	0.656	M3 Ca	0.655
18	M3 Mg	0.629	Sat. at 0	0.635	Nd	0.646
19	M3 Ca	0.626	M3 Ca	0.626	Ca	0.646
20	Cu	0.600	M3 Mn	0.619	pF4.2	0.642
21	pF4.2	0.598	Cu	0.589	Ta	0.637
22	Sand	0.593	Ex. Ac.	0.583	Ce	0.630
23	Clay	0.592	Sand	0.578	M3 Mg	0.628
24	Silt	0.588	Mn	0.576	Ni	0.623
25	Se	0.562	Clay	0.575	Ti	0.621
26	Th	0.559	Sr	0.569	La	0.621
27	M3 Mn	0.557	Ni	0.567	Cr	0.620
28	V	0.554	M3 B	0.557	Cu	0.619
29	Nd	0.546	Ce	0.550	Mn	0.618
30	Ta	0.545	M3 Fe	0.547	V	0.608

## Data Availability

Not applicable.

## Appendix A

**Table A1:** Descriptive statistics of properties in the “Texture” group.

Property	Units	Mean	SD	Min.	Max.	*IQR*	Skewness
Sand	%	84.97	14.32	0.44	100.00	9.50	−2.71
Silt	%	8.30	5.94	0.00	33.92	5.71	1.65
Clay	%	6.73	9.51	0.00	82.15	5.35	4.00

**Table A2:** Descriptive statistics of properties in the “Water” group.

Property	Units	Mean	SD	Min.	Max.	*IQR*	Skewness
Sat. at 0	g/g	0.41	0.14	0.09	1.10	0.14	1.21
pF2.0	g/g	0.27	0.12	0.02	0.71	0.17	0.58
pF2.5	g/g	0.23	0.12	0.01	0.67	0.18	0.69
pF4.2	g/g	0.15	0.11	0.00	0.58	0.15	1.10
B. Den.	g/cm^3^	1.03	0.16	0.58	1.41	0.23	−0.02

**Table A3:** Descriptive statistics of properties in the “CN” group.

Property	Units	Mean	SD	Min.	Max.	*IQR*	Skewness
Total N	%	0.08	0.08	0.00	0.58	0.07	2.46
Total C	%	1.21	1.31	0.09	11.19	1.11	2.69
SON	%	0.08	0.08	0.00	0.56	0.06	2.46
SOC	%	1.14	1.26	0.09	10.40	1.13	2.69

**Table A4:** Descriptive statistics of properties in the “Elements” group.

Property	Unit	Mean	SD	Min.	Max.	*IQR*	Skewness
Na	mg/kg	23,184.64	8764.41	9568.58	92,079.60	7023.58	3.60
Al	mg/kg	30,499.97	18,155.75	824.40	120,089.47	25,022.70	0.63
Cl	mg/kg	214.66	1210.23	8.90	28,193.55	111.00	21.85
K	mg/kg	11,055.74	12,002.48	147.40	97,368.89	14,449.81	2.29
Ca	mg/kg	7558.74	24,037.62	55.30	426,430.90	4772.05	11.01
Ti	mg/kg	3907.00	4195.35	2.10	56,099.97	2970.08	5.02
V	mg/kg	28.49	39.99	1.00	357.90	28.90	3.61
Cr	mg/kg	61.13	84.01	1.00	1148.79	55.30	6.08
Mn	mg/kg	390.29	451.06	5.40	3863.57	408.00	2.61
Fe	mg/kg	26,233.94	27,297.12	74.30	181,690.38	26,458.38	2.43
Ni	mg/kg	19.40	27.15	0.30	286.00	20.70	4.43
Cu	mg/kg	14.91	15.06	0.70	128.40	15.60	2.67
Zn	mg/kg	25.66	27.97	0.90	349.00	27.40	4.38
Ga	mg/kg	7.18	5.95	0.20	28.20	9.20	0.95
Se	mg/kg	886.63	16.80	877.20	1000.00	3.20	6.47
Rb	mg/kg	54.33	47.21	1.30	351.30	60.10	1.64
Sr	mg/kg	85.46	155.68	0.40	1984.91	77.40	5.57
Y	mg/kg	10.73	11.41	0.20	105.40	11.20	2.76
Zr	mg/kg	65.63	114.80	19.70	950.41	5.10	4.03
Ba	mg/kg	1788.02	2985.26	16.50	42,787.14	2237.29	6.50
La	mg/kg	1974.23	4428.23	1.90	65,385.60	1506.81	8.13
Ce	mg/kg	48.05	48.38	1.20	413.30	58.30	1.96
Pr	mg/kg	2.04	3.41	0.50	29.70	0.60	4.00
Nd	mg/kg	8.14	8.80	0.90	56.60	9.40	2.15
Sm	mg/kg	148.84	737.65	0.60	9806.80	19.20	8.40
Hf	mg/kg	2.90	4.37	0.20	52.90	3.30	4.36
Ta	mg/kg	4.54	6.68	0.10	64.30	4.10	5.24
W	mg/kg	0.93	2.24	0.20	30.70	0.30	7.25
Hg	mg/kg	1.97	4.85	0.10	36.80	0.40	4.05
Pb	mg/kg	37.51	67.18	0.40	686.80	34.60	5.63
Bi	mg/kg	1.18	3.06	0.10	37.50	0.90	6.10
Th	mg/kg	39.82	91.74	0.20	818.50	29.20	4.89

**Table A5:** Descriptive statistics of properties in the “Mehlich” group.

Property	Units	Mean	SD	Min.	Max.	*IQR*	Skewness
EC	dS/m	0.13	0.31	0.01	3.88	0.08	8.11
Ex. Ac.	cmol/kg	0.50	0.58	0.05	4.21	0.52	2.39
Ex. Bas.	cmol/kg	14.03	23.63	0.05	186.95	10.14	3.40
M3 Al	mg/kg	842.34	488.75	3.00	3041.00	660.25	1.00
M3 B	mg/kg	0.48	1.71	0.00	23.55	0.35	8.82
M3 Ca	mg/kg	2096.97	4126.19	0.00	35,200.00	1332.25	4.09
M3Cu	mg/kg	1.74	2.11	0.00	23.70	1.86	4.65
M3 Fe	mg/kg	116.00	92.59	1.26	782.00	88.28	2.76
M3 K	mg/kg	167.05	251.24	5.50	3432.00	128.70	6.02
M3 Mg	mg/kg	309.07	412.41	0.00	4740.00	300.32	4.19
M3 Mn	mg/kg	101.61	104.46	1.05	592.00	116.33	1.67
M3 Na	mg/kg	130.06	998.77	0.00	23,000.00	29.75	21.22
M3 P	mg/kg	11.77	28.99	0.00	396.00	7.08	7.86
M3 S	mg/kg	30.03	212.38	0.62	3940.00	8.25	15.07
M3 Zn	mg/kg	1.46	1.61	0.00	23.80	0.96	6.42
pH		6.19	1.09	3.61	9.72	1.33	0.68
PSI		84.88	89.50	−116.90	452.89	88.27	1.80
